# Potential pathogenic roles of ferroptosis and cuproptosis in cadmium-induced or exacerbated cardiovascular complications in individuals with diabetes

**DOI:** 10.3389/fendo.2024.1461171

**Published:** 2024-10-02

**Authors:** Saman Saedi, Yi Tan, Sara E. Watson, Kupper A. Wintergerst, Lu Cai

**Affiliations:** ^1^ Department of Animal Science, College of Agriculture, Shiraz University, Shiraz, Iran; ^2^ Pediatric Research Institute, Department of Pediatrics, University of Louisville School of Medicine, Louisville, KY, United States; ^3^ Wendy Novak Diabetes Institute, Norton Children’s Hospital, Louisville, KY, United States; ^4^ Department of Pharmacology and Toxicology, University of Louisville School of Medicine, Louisville, KY, United States; ^5^ Division of Endocrinology, Department of Pediatrics, University of Louisville, Norton Children’s Hospital, Louisville, KY, United States; ^6^ The Center for Integrative Environmental Health Sciences, University of Louisville School of Medicine, Louisville, KY, United States; ^7^ Department of Radiation Oncology, University of Louisville School of Medicine, Louisville, KY, United States

**Keywords:** cadmium, cuproptosis, cardiovascular diseases, diabetic cardiomyopathy, diabetes, ferroptosis

## Abstract

Diabetes and its complications are major diseases that affect human health. Diabetic cardiovascular complications such as cardiovascular diseases (CVDs) are the major complications of diabetes, which are associated with the loss of cardiovascular cells. Pathogenically the role of ferroptosis, an iron-dependent cell death, and cuproptosis, a copper-dependent cell death has recently been receiving attention for the pathogenesis of diabetes and its cardiovascular complications. How exposure to environmental metals affects these two metal-dependent cell deaths in cardiovascular pathogenesis under diabetic and nondiabetic conditions remains largely unknown. As an omnipresent environmental metal, cadmium exposure can cause oxidative stress in the diabetic cardiomyocytes, leading to iron accumulation, glutathione depletion, lipid peroxidation, and finally exacerbate ferroptosis and disrupt the cardiac. Moreover, cadmium-induced hyperglycemia can enhance the circulation of advanced glycation end products (AGEs). Excessive AGEs in diabetes promote the upregulation of copper importer solute carrier family 31 member 1 through activating transcription factor 3/transcription factor PU.1, thereby increasing intracellular Cu^+^ accumulation in cardiomyocytes and disturbing Cu^+^ homeostasis, leading to a decline of Fe–S cluster protein and reactive oxygen species accumulation in cardiomyocytes mitochondria. In this review, we summarize the available evidence and the most recent advances exploring the underlying mechanisms of ferroptosis and cuproptosis in CVDs and diabetic cardiovascular complications, to provide critical perspectives on the potential pathogenic roles of ferroptosis and cuproptosis in cadmium-induced or exacerbated cardiovascular complications in diabetic individuals.

## 1 Introduction

Diabetes is an endocrine system disease caused by a chronic hyperglycemic state. Indeed, diabetes is characterized by high blood glucose levels that result from insulin deficiency, in the context of β- cell dysfunction, insulin resistance or both (1). This chronic metabolic disorder is one of the most common and fastest-growing diseases worldwide, projected to affect 693 million adults by 2045 (2). Vascular complications of both the macrovascular system (cardiovascular disease (CVD) and microvascular system (diabetic kidney disease, diabetic retinopathy, and neuropathy) are the leading cause of morbidity and mortality in individuals with diabetes (3). Additionally, studies have shown that CVD is the major contributor to morbidity and mortality in individuals with type 2 diabetes (T2D), with a risk that is doubled in patients with diabetes compared with those without diabetes (4). These studies show diabetes acts as an independent risk factor for several forms of CVDs such as coronary artery diseases, heart failure (HF), cardiomyopathy, arrhythmia, and peripheral artery disease (5). To make matters worse, when patients with diabetes develop clinical CVDs, they sustain a worse prognosis for survival than do CVD patients without diabetes (6).

Diabetes and its cardiovascular complications are complex, multifactorial conditions with both main environmental and genetic components. Major risk factors for cardiovascular complications among individuals with diabetes include the presence of other microvascular complications, as well as BMI, age, sex, blood pressure, smoking status, and heavy metals (7–9). Cadmium is a toxic heavy metal that has been associated with hypertension, promotion of atherosclerosis, and impaired cardiac function (10). Experimental studies indicated that cadmium exposure is associated with increased risk of HF and damages the cardiomyocyte (11, 12). In addition, epidemiological studies suggested that higher cadmium exposure is associated with disruption of pancreatic islet function and an increased prevalence of diabetes (13–15). Although, it has been indicated that cadmium exacerbates cardiovascular complications of diabetic mice (9), the relationship between cadmium exposure to induce or exacerbate cardiovascular complications in individuals with diabetes is not clear.

Ferroptosis and cuproptosis are recently discovered form of regulated cell death triggered by excess iron (Fe) and copper (Cu^2+^), respectively (16, 17). Cuproptosis is highly related to cellular metabolism (18), and ferroptosis plays a critical role in cellular proliferation, and differentiation (16). Ferroptosis and cuproptosis have attracted significant interest due to their association with numerous pathological processes (19, 20). Recent studies have shown that impaired ferroptosis was implicated in the occurrence and development of various CVD (21). On the other hand, the essential role of Cu homeostasis has been shown in diabetic cardiomyopathy (DCM) (22).

Environmental toxicant exposures play a significant role in the development and progression of diabetic complications, particularly in T2D. The interaction between genetic predispositions and environmental factors can influence the clinical presentation of diabetes and its associated complications (23). Cadmium as an environmental toxin is highlighted due to its role in exacerbating complications in diabetic patients, particularly in comparison to other environmental toxins. Cadmium leads to oxidative stress and inflammation, which are known contributors to the progression of diabetic complications (24). Studies have demonstrated that cadmium exposure increases markers of oxidative stress, such as malondialdehyde, while decreasing antioxidant levels in diabetic subjects, thereby worsening diabetic complications (25). Besides, cadmium may disrupt trace element homeostasis such as zinc (Zn), magnesium (Mg), Cu and iron (26–29). These findings indicate that cadmium exposure may induce ferroptosis and cuproptosis mediated by change trace element homeostasis. Besides, previous studies have shown that ferroptosis and cuproptosis are associated with the risk of CVDs and diabetic cardiovascular complications (16, 30–32). However, potential roles of ferroptosis and cuproptosis in cadmium-induced or exacerbated cardiovascular complications in individuals with diabetes are still unknown. And whether there is a relationship between ferroptosis and cuproptosis with cadmium-induced cardiovascular complications in the individuals with diabetes remains unclear. In the current review, we present an overview on the most recent advances exploring the underlying mechanisms of ferroptosis and cuproptosis in CVDs and diabetic cardiovascular complications and provide critical perspectives on the potential pathogenic roles of ferroptosis and cuproptosis during cadmium exposure in cadmium-induced or exacerbated cardiovascular complications in the individuals with diabetes.

## 2 Cadmium and CVD in the individuals with diabetes

### 2.1 Cadmium

Cadmium is a toxic heavy metal harmful to human health, which is released by industrial activities such as leachate from combustion of fossil fuels, mining residues, and human activities including pesticides or naturally present in the environment (33). In the United States, approximately 600 tons of cadmium compounds are produced yearly and 150 tons are imported from other countries (34). Cadmium has a long biological half-life (10-30 years) and the average dietary intake of this metal ranges from 8–25 μg per day (35, 36). Cadmium can enter animals’ and humans’ bodies via breathing, food, and drinking water and accumulate in the organism, and cause severe damage to a variety of organs, including the lungs, liver, kidneys, testes, and brain (27, 37, 38). It has been reported that cadmium exposure is correlated with many health disorders such as neurotoxicity, osteoporosis, gastrointestinal injury, cancer, and diabetes (39–43).

### 2.2 Diabetes

Diabetes is a complex metabolic disorder characterized mainly by chronic hyperglycemia, a physiologically abnormal condition represented by continued enhanced blood glucose levels (1). In 2019, the number of adults with diabetes is estimated to be around 463 million, which corresponds to 9.3% of the total adult world population. By 2030, this number is estimated to rise to 578 million, representing 10.2% of the world’s total adult population, and further increase to 700 million by 2045, which represents 10.9% of the total world adult population (44). There are three main types of diabetes: type 1 diabetes (T1D), type 2 diabetes (T2D), and gestational diabetes during pregnancy. T1D is thought to be caused by an autoimmune reaction characterized by the destruction of the pancreatic islets of beta cells, leading to insufficient insulin secretion. Approximately 5-10% of the people who have diabetes have T1D. However, T2D accounts for more than 90% of all cases (45, 46). Diabetes and its complications are complex, and both major environmental and genetic factors participate in its etiopathogenesis (1). T2D has been more commonly associated with increasing age, family history of diabetes, obesity, physical inactivity, and adoption of modern lifestyle, but these factors are complex and remain largely unspecified. Nonetheless, unlike T1D, this disease is not associated with genes involved in the immune response including autoimmunity and consequently there is no immune-mediated destruction of pancreatic β-cells (47, 48).

### 2.3 Diabetic cardiovascular complications

Diabetes not only causes microangiopathy (associated with the three major diabetic complications, namely diabetic nephropathy, retinopathy, and neuropathy) but is also a major risk factor for macroangiopathy such as CVDs (49, 50). It has been indicated that diabetes can lead to multiple cardiovascular complications, including coronary artery disease, HF, and cardiac hypertrophy (51). CVDs are the leading cause of death worldwide, and they disproportionally affect people living in different communities and environments. Yearly, CVDs cause immense economic and health burdens in the United States and worldwide. In 2020, approximately 19 million deaths were attributed to CVDs, which was an increase of 18.7% since 2010 (52). Depending on the diabetes subtype and CVD complications for example, myocardial infarction, coronary heart disease, HF or stroke, individuals with diabetes have anywhere from a twofold to tenfold increased risk of CVD complications compared to individuals without diabetes (49, 53, 54). Despite the well-known increased risk of CVDs among individuals with diabetes, the pathophysiology linking the two conditions is poorly understood.

DCM is a specific diabetes-induced CVD that refers to a group of diseases that affect the heart muscle, leading to cardiac structure remodeling and dysfunction. Cell death, especially cardiomyocyte death, plays a critical role in the development of cardiomyopathy. When cardiomyocytes die, either by necrosis (uncontrolled cell death) or apoptosis (programmed cell death), there is a loss of contractile units, leading to impaired heart function. This occurs because cell death is followed by energy imbalances, chronic inflammation, and oxidative stress, all of which play crucial roles in the development and progression of cardiac dysfunction, i.e., cardiomyopathy (55–57). On the other hand, oxidative stress increases in patients with diabetes and animal models of diabetes (58). Indeed, oxidative stress and inflammation play a key role in the pathogenesis and progression of diabetes-induced CVD, where the enhanced expression of inflammatory proteins or cytokines such as oxidative stress-related proteins or C-reactive protein are shown to serve as biomarkers for the occurrence of cardiovascular complications (59).

### 2.4 Cadmium induces CVDs

Recent studies have highlighted the risks of chronic exposure to environmental toxicants, including heavy metals such as inorganic arsenic, lead, and cadmium in CVDs. Despite the fact that the exact effects of cadmium on the cardiovascular system are controversial, studies have shown that it can affect the cardiovascular system at extremely low doses. An *in vitro* study showed that a dose of cadmium less than toxic concentrations can trigger pathological changes in the vessel walls (60). Besides, chronic cadmium exposure has been associated with promotion of atherosclerosis, hypertension, and impaired cardiac function, as well as an increased risk of cardiovascular mortality (10, 61, 62). In addition, CVDs, stroke, coronary heart disease, and peripheral arterial disease increase due to cadmium exposure (63).

The underlying mechanisms by which cadmium can induce CVDs, cardiomyopathy, and coronary heart disease are still unclear, and further studies are needed to elucidate this issue. However, it has demonstrated that cadmium can disrupt endothelial cells integrity and induces cadmium-mediated endothelial cell death. The formation of gaps between the endothelial cells allows cadmium to diffuse from the bloodstream into the media layer (64). Cadmium is mainly retained in the smooth muscle cells after transport across the endothelial cells and can affect smooth muscle cells. Its effects on smooth muscle cells include cytotoxic effects, interactions with ion homeostasis and calcium ion flux, and stimulation of smooth muscle cell proliferation at low cadmium concentrations, leading to subsequent lipid accumulation in vessel walls and alteration of lipid profiles toward a more atherogenic state (65, 66). Cadmium accumulation also increases multiple important pro-inflammatory cytokines such as interleukin (IL)-6, IL-8, IL-1β, and tumor necrosis factor α (TNF-α) (67), which may play a critical role in atherosclerosis (68, 69). Moreover, exposure to cadmium alters lipid metabolism and decreases nitric oxide release from endothelial cells, leading to atherosclerosis, stroke, and hypertension (70).

## 3 Potential roles of cadmium-exacerbates cardiovascular complications in individuals with diabetes

**Figure 1:**
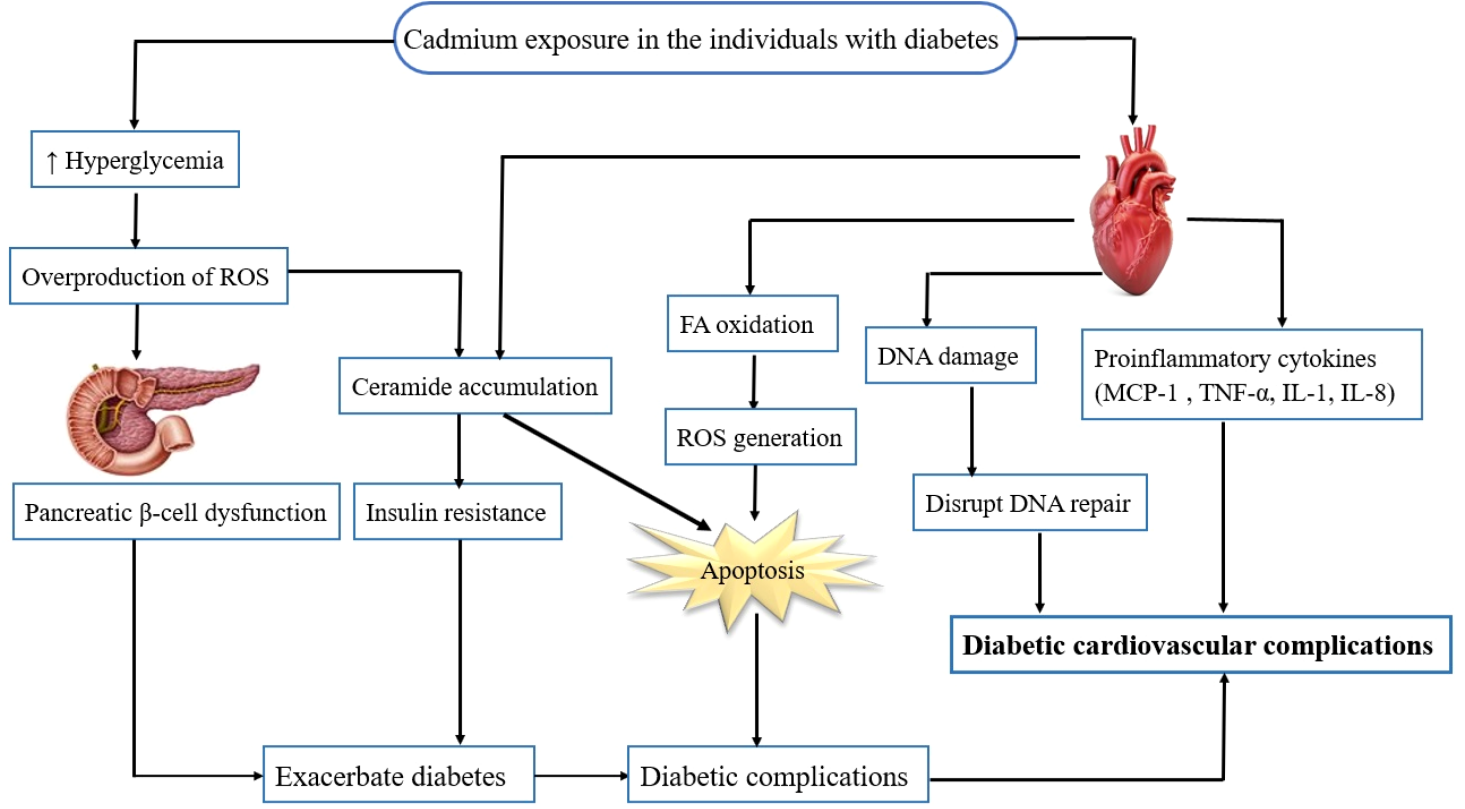
Potential role of cadmium in exacerbating cardiovascular complications in individuals with diabetes. Cadmium exposure in individuals with diabetes results in hyperglycemia, ceramide accumulation, and an increase in the FFA oxidation, proinflammatory cytokines as well as generation of ROS. The ROS activate several pathways involved in the pathogenesis of diabetic cardiovascular complications, including inflammation, disrupting DNA repair, oxidative stress, apoptosis, and cell death. FFA, free fatty acid; ROS, reactive oxygen species.

Cardiovascular disease remains the leading cause of mortality and morbidity in individuals with diabetes. Recent studies have presented the risk of cardiotoxic effects of exposure to multiple metals particularly under diabetic or metabolic conditions (71–73). In one of these studies, Long et al. (72) demonstrated inverse associations between selenium and zinc incident CVD risk in patients with T2D. However, evidence for the association between heavy metal levels, especially cadmium and the risk of CVDs among individuals with T2D is limited. A recent study has presented that exposure to cadmium is significantly associated with higher risk of CVD mortality among patients with T2D (74). Animal studies have shown the effect of cadmium exposure on cardiac glucose metabolism and lipid accumulation in male Wistar rats. This study has indicated that cadmium exposure induces cardiac glucometabolic dysregulation and lipid accumulation independent of pyruvate dehydrogenase activity which could reduce cardiac efficiency (75). Besides, Xiuxiu Liu (9) has reported that cadmium exacerbates DCM development in mice. Altogether, potential roles of cadmium in exacerbating cardiovascular complications in the individuals with diabetes are described below (**Figure 1**).

### 3.1 Oxidative stress

Based on the above discussion, diabetes is associated with cadmium exposure and cadmium is also a risk factor for cardiac oxidative damage. Cadmium-induced cardiac oxidative stress is mediated by free radicals generation and subsequent production of reactive oxygen species (ROS) such as superoxide anion free radical (O_2_.^−^), hydrogen peroxide (H_2_O_2_) as well as hydroxyl free radical (HO.) in the heart (76, 77). Enhanced production of ROS can overwhelm cells’ intrinsic antioxidant defenses, and result in oxidative stress, lipid oxidation, cell cycle progression, DNA damage, and apoptosis (78). Cadmium exposure provides cardiac oxidative damage; therefore, this heavy metal may also exacerbate cardiac dysfunction or CVDs in individuals with diabetes.

On the other hand, cadmium exposure is reported to induced hyperglycemia and oxidative status, leading to pancreatic β-cell dysfunction and progression of diabetes (79, 80). Hyperglycemia induces overproduction of some ROS like O_2_.^−^ by the mitochondrial electron transport chain. It could be the first and key event in the activation of all other pathways involved in the pathogenesis of diabetic complications (81, 82) such as oxidative stress, inflammation, and CVDs (83). Additionally, studies have demonstrated that ROS generation under various pathological conditions such as cadmium exposure triggers ceramide accumulation in diabetic myocardium (83, 84). Ceramide accumulation has been proposed to be involved in insulin resistance, triggering cardiomyocyte apoptosis, cardiac arrhythmias, and CVDs (85, 86).

Generally, the heart uses fatty acid (60—80%) or glucose oxidation to produce adenosine triphosphate (ATP). Cadmium can further increase fatty acid (FA) oxidation and inhibit glucose oxidation in the heart under diabetic condition. FA oxidation decreases ATP and enhances ROS production in the mitochondria. A decrease in ATP and increased ROS production in the mitochondria due to cadmium exposure in individuals with diabetes leads to cardiovascular complications (77, 87).

### 3.2 Inflammation/signaling pathways changes

Cadmium disorders the arrangement of myocardial fibers and accelerates the fibrosis on myocardial fibers in diabetic mice. Furthermore, cadmium increases the level of transforming growth factor β1 (TGF-β1) in diabetic mice (9). TGF-β1 may contribute to cardiac dysfunction and induce DCM (88). On the other hand, cadmium exposure raises proinflammatory cytokines such as TNF-α, IL-1, and monocyte chemoattractant protein-1 (MCP-1) in diabetic mice (9). Rise of TNF-α, IL-1, and IL-8, levels in the serum and pancreas may affect inflammation in CVDs (89). MCP-1 plays a causative role in experimental DCM, and MCP-1 deficiency in animal models can attenuate HF (90–92). However, evidence to confirm the role of cadmium in exacerbating the risk of diabetic cardiovascular complications is limited, so more studies are needed to understand its underlying mechanisms.

### 3.3 Epigenetic effect

Other potential biological mechanisms for the effects of heavy metals, particularly cadmium, on the cardiovascular system include altered regulation of endocrine and endothelial vascular functions, as well as epigenetic pathways (93, 94). Some heavy metals can form covalent bonds with sulfhydryl groups of proteins due to their electron-sharing properties (95, 96). The binding to glutathione leads to depletion of its levels, resulting in an increase in the intracellular concentration of ROS. The consequences of this process include damage to cell membranes, promotion of lipid peroxidation, DNA damage, oxidation of amino acids in proteins leading to changes in their structure and function, and inactivation of enzymes (95). Increased oxidative stress and ROS generation may lead to the formation of gene products that cause cellular damage and loss of activity of DNA repair pathways, thereby contributing to the pathogenesis of diabetic cardiovascular complications (83, 97, 98).

### 3.4 Disturbing essential homeostasis

Interference with essential metal homeostasis, induction of oxidative stress and apoptosis by multifactorial mechanisms are considered as the most influential forms of cadmium toxicity in humans (94). Chronic exposure to cadmium results in several disorders through disrupt the homeostasis of trace elements such as Zn, Mg, Cu, and iron (26–29). For instance, cadmium displaces Zn in many sulfur-containing proteins, which leads to their dysfunction and the subsequent disruption of numerous biological processes (29). Animal study by (27) has demonstrated that early exposure to cadmium remarkably affected essential metals homeostasis such as Zn, Mg, Cu, and Fe levels in the kidney, liver, and heart in offspring. Cadmium can produce ROS by indirectly displacing an endogenous Fenton metal (e.g., Fe^2+^) from proteins, thus increasing the amount of free redox-active metals (99). Besides, excessive ROS production can lead to mitochondrial membrane depolarization, macromolecule oxidation, and apoptosis (99). Therefore, cadmium can disturb essential homeostasis by altering essential metals’ homeostasis and inducing and exacerbating different types of cell death such as apoptosis and necrosis. However, in the continuation of current review, we introduce other types of cell death such as ferroptosis and cuproptosis and provide potential pathogenic roles of ferroptosis and cuproptosis during cadmium exposure in cadmium-induced or exacerbated cardiovascular complications in the individuals with diabetes.

## 4 Ferroptosis

**Figure 2:**
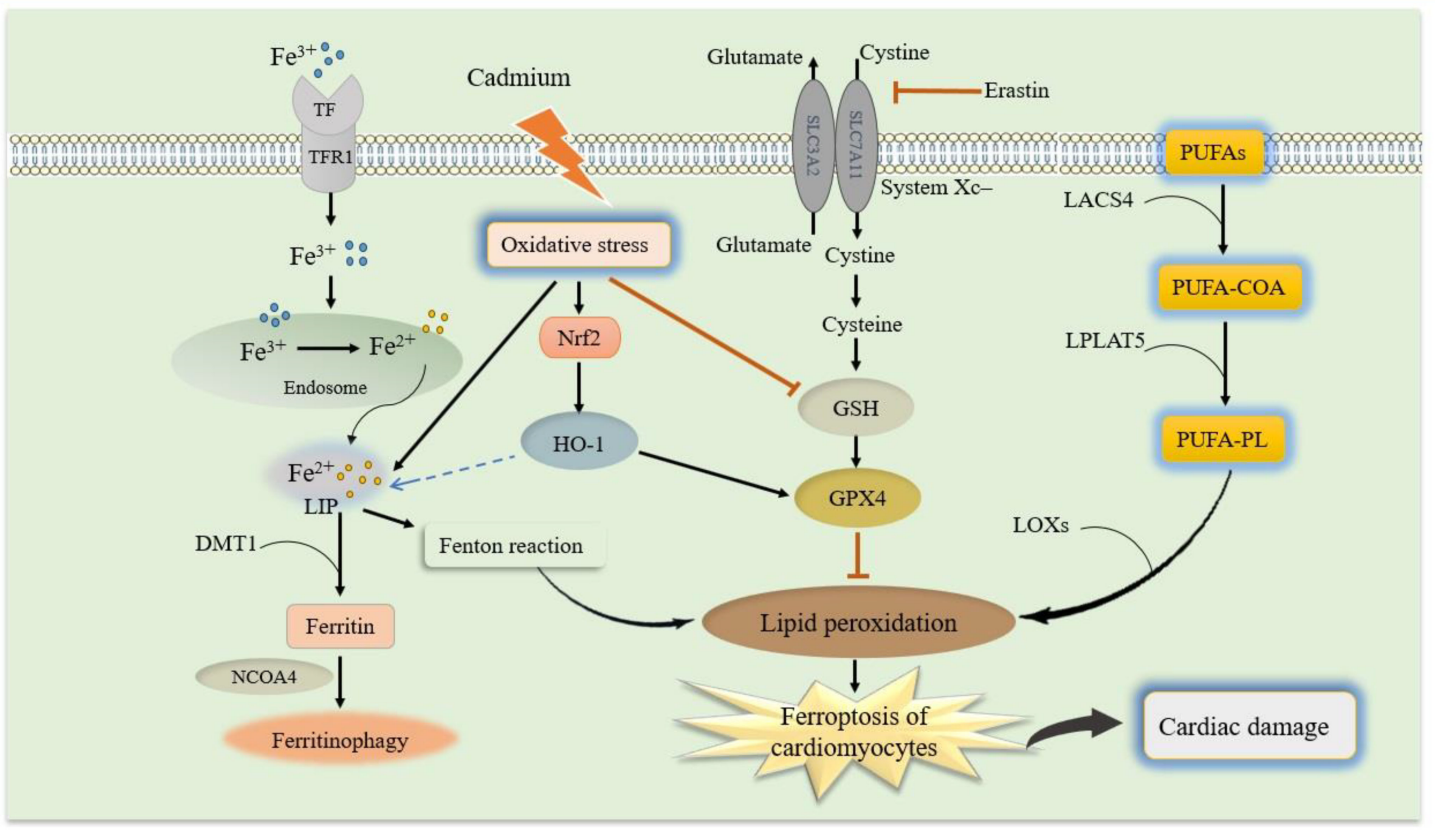
Schematic model of ferroptosis and molecular mechanisms of cadmium-exacerbated ferroptosis in diabetic cardiomyocytes. Molecular mechanisms of ferroptosis and related signaling pathways, which mainly include iron metabolism, GSH and GPX4, and lipid peroxidation. (1) Iron uptake via the transferrin receptor 1 (TfR1) or degradation of ferritin iron stores increases the labile iron pool, thereby cells are susceptible to ferroptosis via the formation of lipid hydroperoxides through the Fenton-like reaction. (2) Cystine-glutamate antiporter system Xc- regulates cysteine and GSH availability. GPX4 utilizes GSH to reduce lipid hydroperoxides, thereby preventing lipid peroxidation chain reactions. (3) The PUFA-PLs that are derived from PUFA-CoA by LPCAT3 and PUFA by ACSL4, respectively, are the main causative of lipid peroxidation. Cadmium exposure can cause oxidative stress in the diabetic cardiomyocytes, leading to iron accumulation, GSH depletion, lipid peroxidation, and finally exacerbate ferroptosis and disrupt the cardiac. In addition, Nrf2 up-regulation due to cadmium-induced oxidative stress can increase HO-1 expression to regulate iron hemostasis. GSH, reduced glutathione; GPX4, glutathione peroxidase 4; system Xc-, cystine–glutamate antiporter; PUFAs, polyunsaturated fatty acids; PUFA-CoA, PUFA coenzyme A; PUFA-PL, phospholipid PUFA; Nrf2, nuclear factor erythroid 2-related factor 2; HO-1, heme oxygenase.

Ferroptosis is a novel non-apoptotic form of regulated cell death (100). Ferroptosis is induced by small molecules that inhibit glutathione (GSH) biosynthesis or glutathione peroxidase 4 (GPX4), and is characterized by iron-dependent accumulation of ROS and consumption of polyunsaturated fatty acids (PUFAs) in the plasma membrane (101). Ferroptosis differs from necroptosis, apoptosis, autophagy, and other types of cell death in terms of morphological characteristics and functions (102). The signaling pathway involved in ferroptosis includes multiple interconnected molecular events that key players in this pathway include iron metabolism, lipid metabolism, and antioxidant systems (30, 103). During the process of ferroptosis, the activity of cystine–glutamate antiporter (system Xc-) is inhibited, namely the amount of cystine entering into cells and glutamate transporting out of cells decreases. The lipid peroxide accumulates (103, 104). The production of lipid peroxides is iron-dependent. Iron accumulation in lipid oxidation facilitates the overproduction of lipid ROS. Then, reduced lipid peroxide scavenging due to inhibition of lipid antioxidants (e.g., GPX4) leads to ferroptosis (103, 105). Besides, other molecules and processes also contribute to the regulation of ferroptosis. The nicotinamide adenine dinucleotide phosphate oxidase (NOX) family of enzymes, such as NOX1 and NOX2, generate ROS that can contribute to lipid peroxidation and ferroptosis (106) as shown in **Figure 2**.

### 4.1 Ferroptosis in diabetes

Ferroptosis has been implicated to play a pathogenic role in several disorders extending various systems, including liver disease, lung disease, cardiovascular disease, neurodegenerative disorders, cancers, kidney injury, gastrointestinal disease, and pancreas dysfunction (107). Since ferroptosis is regulated by the iron-dependent accumulation of lipid peroxides and subsequent oxidative damage, it has been suggested that ferroptosis may play a role in the pathogenesis of diabetes (108, 109). Excessive iron stores have been demonstrated to be associated with the development of diabetes, and ferritin levels are increased in T2D (110). Elevated ferritin in diabetes patients led to hyperglycemia (111). Besides, other iron metabolism indicators such as hepcidin could also be related to diabetic pathogenesis (110). Substantial researches have called that iron deficiency and excess may affect glucose regulation (112), and high glucose can cause a rise to iron overload (113), which is known to trigger ferroptosis. Meanwhile, under iron overload condition, oxidative stress can induce insulin resistance (114).

Pancreatic islet β-cells are susceptible to ferroptosis. Some studies indicate that pancreatic β-cells are susceptible to oxidative stress because they express a low level of antioxidant enzymes such as superoxide dismutase, GSH peroxidase, and catalase (115); therefore, ROS is prone to accumulate in the pancreatic islet β-cells. Accumulation of ROS in the pancreatic islet β-cells can trigger many forms of deterioration, including ferroptosis (105). Pancreatic islet β-cells function is closely associated with diabetes, and insufficient insulin secretion caused by pancreatic β-cells failure contributes to hyperglycemia. Hyperglycemia may inflict solute carrier family 7 member 11 and solute carrier family 3 member 2 impairment, leading to system Xc– dysfunction in the molecular mechanism of ferroptosis (116). Increased cellular iron alters the expression of genes involved in β-cell function and causes pancreatic β-cell dysfunction (117). Nutrient-deprivation autophagy factor-1 (NAF-1), is a class of iron-sulfur proteins that regulates mitochondrial iron levels (118). Interestingly, suppressed expression of NAF-1 in INS-1E pancreatic β-cell results in the appearance of ferroptosis-like features characterized by enhanced lipid peroxidation, and decreased expression of GPX4 (119). Krummel et al. have implicated that β-cells may have a high sensitivity to ferroptosis and confirmed that GPX4 distributes throughout the β-cell to a large extent, eliminating GPX4 induces ferroptosis (120). Altogether, ferroptosis features including upregulation in transferrin expression, elevated lipid peroxidation, and reduction in GPX4 have been observed in INS-1 E pancreatic β-cell, following the suppressed expression of NAF-1, which is a protein that regulates mitochondrial iron levels (107). A study showed remarkably reduced glucose-stimulated insulin secretion (GSIS) capacity in human islet β-cells when they were treated with the ferroptosis inducer erastin *in vitro* (121). Conversely, pretreatment with a ferroptosis inhibitor, Fer-1, rescued the damage to GSIS (121). On the other hand, inhibition of ferroptosis using specific inhibitor Fer-1 as the first ferroptosis inhibitor effectively improves the viability and functionality of pancreatic islets (122). These data indicate that ferroptosis as a new mechanism of cell death, plays an important role in maintaining homeostasis in the pancreatic islet cells, and may help to develop novel treatments for diabetes.

### 4.2 Ferroptosis in diabetic cardiovascular complications

Diabetes onset leads to a wide spectrum of macrovascular and microvascular disorders that affect the majority of the body. Diabetes is a high-risk factor to contribute CVDs, including atherosclerosis, myocardial ischemia/reperfusion injury in diabetes, DCM, and HF (123). Recent evidence illustrates that the development of diabetes complications particularly diabetic cardiovascular complications is closely related to ferroptosis (107). DCM is a group of myocardial diseases in patients with diabetes, which is distinguished by early onset of ventricular diastolic dysfunction and late onset of ventricular systolic dysfunction, cardiac hypertrophy, and fibrosis (124). The pathogenesis of DCM is primarily due to impaired cardiac energy metabolism and mitochondrial abnormalities, which consequently lead to ROS-mediated oxidative stress, and cardiac inflammation, all of which are associated with the same pathogenic risk factors for ferroptosis (108). Zang et al. demonstrated a potential increase in ferroptosis in the hearts of mice with T1D through increased iron deposition and cardiac 4-hydroxy-2-nonenal, a biomarker of lipid peroxidation for ferroptosis (125). Meanwhile, increased expression of cyclooxygenase 1 and fatty acid coenzyme A ligase proteins in the ferroptosis signaling pathway was observed in the hearts of rabbits with T1D, along with decreased GPX4 protein levels (126). Lipid peroxidation and reduced lipid peroxide scavenging due to inhibition of lipid antioxidants (e.g., GPX4) are pathogenic risk factors for ferroptosis.

Nuclear factor erythroid 2-related factor 2 (Nrf2) is a transcription factor controlling the expression of many ferroptosis-related genes such as GPX4 (127). Nrf2 and its target genes have an inhibitory effect on ferroptosis and act as a major regulator of cell redox state and detoxification to prevent high glucose-induced oxidative damage in DCM (128). Recent studies have shown that activation of Nrf2 reduced oxidative damage induced by high glucose in cultured cardiomyocytes and prevented the development of DCM in animal models (129, 130). Therefore, inactivating Nrf2 could mediate antioxidant defense and impair iron metabolism (125). Nevertheless, an interesting study demonstrated that Nrf2 has destructive effects on the heart by promoting ferroptosis during myocardial autophagy deficiency (125). However, targeting Nrf2 and its related targets remains a viable approach to prevent or treat DCM by regulating ferroptosis, which undoubtedly deserve further studies.

Myocardial ischemia is more likely to occur in those with diabetes, leading to myocardial vulnerability (131). Under diabetic conditions, oxidative stress and programmed cell death turn down AMP-activated protein kinase (AMPK) expression, leading to a higher level of NOX, whose main function is to supply ROS (132). Endoplasmic reticulum stress (ERS) is a cellular response to endoplasmic reticulum (ER) dysfunction and can be triggered by ROS, which is produced by the interaction between iron ions and NOX during ferroptosis (123). Erastin could aggravate ERS and cell injury by stimulating ferroptosis in high-glucose hypoxia reoxygenation group cell model. Meanwhile, inhibition of ERS could alleviate ferroptosis and cell injury. Also demonstrated is that suppressing ferroptosis may alleviate ERS, which was triggered by transcription factor 4- CCAAT-enhancer-binding protein homologous protein pathway and delay the progression of diabetic myocardial ischemia/reperfusion injury (133).

### 4.3 Cadmium and ferroptosis in diabetic cardiovascular complications

Several studies have shown that exposure to environmental pollutants such as arsenic and cadmium is closely associated with ferroptosis (134, 135). Cadmium exposure causes pancreatic β-cells dysfunction by disrupting lipid metabolism and inducing lipid accumulation in pancreatic β-cells (136). Cadmium can also cause abnormal iron metabolism and interfere with iron homeostasis (**Figure 2**). Hong et al. suggested that cadmium exposure can lead to iron accumulation, GSH depletion, GPX4 reduction, lipid peroxidation, mitochondrial membrane potential loss, and ultrastructural damage at the mitochondrial level by activating the Ager/Pkc/p65 pathway. Therefore, cadmium can induce ferroptosis of pancreatic β-cells by activating the GPX4/Ager/p65 pathway (137), exacerbating diabetes and its complications. On the other hand, high glucose levels in individuals with diabetes can induce ferroptosis in cardiomyocytes as a complication of diabetes (126).

Cardiomyocytes are rich in mitochondria and serve as the main source of ROS in the heart (138). Exposure to stressful conditions like heavy metals causes activation of lipid peroxidation in the heart (139, 140). Cadmium toxicity also inhibits mitochondrial function in cardiomyocytes by inactivating respiratory chain enzymes which results in the induction of ferroptosis (141). Moreover, cadmium interacts with endogenous and exogenous antioxidants and compromises the redox potential of the cells, which causes a series of complications such as lipid peroxidation of membranous structures, DNA damage–associated genotoxicity, and proteotoxicity associated ER-stress (142, 143). Therefore, this cardiac pathogen induces common conditions such as iron overload in cardiomyocytes, mitochondrial dysfunction, oxidative stress, and lipid peroxidation which lead to ferroptosis and CVDs (139).

Nrf2/heme oxygenase (HO-1) signaling pathway, as a critical signaling pathway in the oxidative stress response, is involved in anti-inflammatory, antioxidant, and other processes. Recent studies suggest that ferroptosis plays an indispensable role in DCM through the Nrf2/HO-1 pathway, and the activation and upregulation of Nrf2 can increase GPX4 and HO-1 expression to alleviate DCM (144). Another major feature of ferroptosis is an increase in intracellular free iron due to a disturbance in iron metabolism. Nrf2 regulates many ferroptosis-related proteins, such as ferritin, transferrin receptor (TfR), ATP-binding cassette subfamily B member 6, ferrochelatase. Nrf2 has also been shown to regulate the metabolism of heme to produce iron by regulating HO-1 and then regulating the concentration of iron in cells to reduce ferroptosis (145). The therapeutic potential of Nrf2 activation was well documented against pathological conditions associated with oxidative stress (146, 147). Additionally, when a cell was exposed to oxidative stress, the expression of the HO-1 mRNA and protein upregulated (145). Oxidative stress is one of the most important pathways of cadmium toxicity (148). Notably, cadmium exposure upregulates Nrf2 mRNA in H9c2 cardiomyocytes, which activate Nrf2 protein (149). Nrf-2-activation-mediated phase 2 enzymes were shown to be protective against toxic effects of cadmium in various cell lines and in mice (149). Cadmium induces cardiac oxidative stress and activates Nrf2 signaling pathway and activation of Nrf2 signaling pathway attenuates cadmium-induced cardiac oxidative stress (150) (**Figure 2**). Besides, activation of AMPK and Nrf2 plays an important role in preventing cardiac ferroptosis and consequently preserving cardiac function in T2D mice (151). Even though Nrf2 activation can be a novel ideal therapeutic strategy for alleviating cardiotoxicity and cardiac ferroptosis caused due to cadmium exposure in diabetic patients, further study is needed to confirm this.

## 5 Cuproptosis

**Figure 3:**
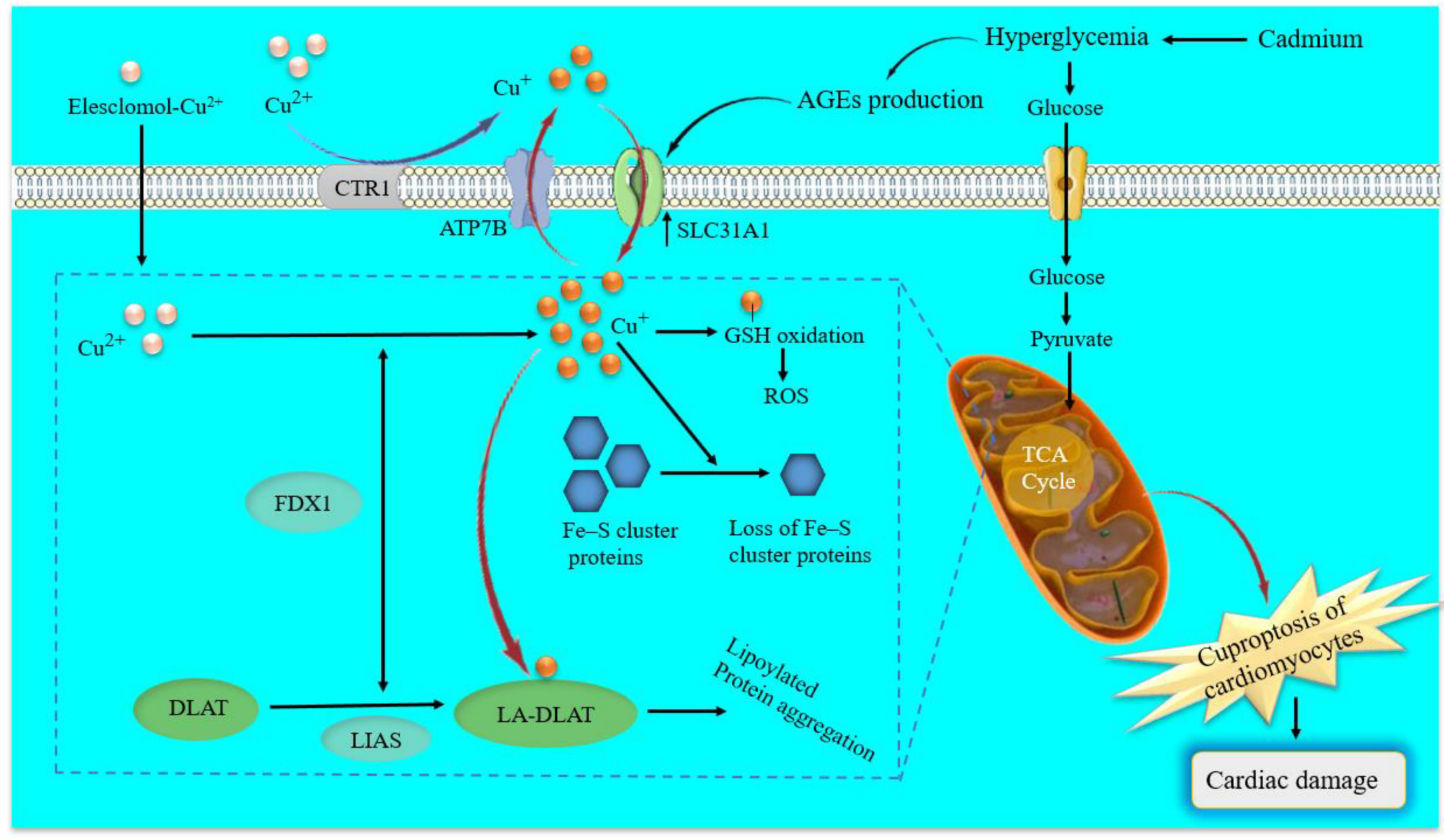
Schematic model of cuproptosis and molecular mechanisms of cadmium-induced cuproptosis in diabetic cardiomyocytes. Elesclomol binds extracellular copper (Cu^2+^) and transports it to intracellular compartments. Besides, copper ion channels SLC31A1 and ATP7B regulate the accumulation of Cu^+^ by mediating the entry and exocytosis of Cu^+^, respectively. Afterward, regulate cuproptosis sensitivity by affecting intracellular Cu^+^ levels. On the one hand, Cu^+^ binds to lipoylated mitochondrial enzymes in the TCA cycle such as DLAT, inducing the aggregation of these proteins. On the other hand, FDX1 as an upstream regulator of protein lipoylation reduces Cu^2+^ to Cu^+^, and with LIAS facilitates the aggregation of mitochondrial proteins and loss of Fe–S cluster proteins. Cadmium-induced hyperglycemia can enhance circulating AGEs. Excessive AGEs in diabetes promote the upregulation of copper importer SLC31A1 through ATF3/SPI1, thereby increasing intracellular Cu^+^ accumulation in cardiomyocytes and disturbing Cu^+^ homeostasis, leading to a decline of Fe–S cluster protein and ROS accumulation in cardiomyocytes mitochondria. ATP7B, ATPase copper transporting beta; SLC31A1, solute carrier family 31 member 1; Fe–S, iron-sulfur; FDX1, ferredoxin 1; TCA, tricarboxylic acid; DLAT, dihydrolipoamide S-acetyltransferase; LA-DLAT, lipoylated DLAT; LIAS, lipoyl synthase; AGEs, Advanced Glycosylation End Products.

The latest research in the field of cellular biology and disease research has discovered a new form of cell death, cuproptosis, which differs from pyroptosis, apoptosis, necroptosis, and ferroptosis (17). This process involves the activation of specific signaling cascades and is associated with changes in mitochondrial enzymes. In particular, the regulation of cuproptosis is based on copper overload, is dependent on mitochondrial respiration and adenosine triphosphate (ATP) production and is closely related to the tricarboxylic acid (TCA) cycle. Indeed, cuproptosis mainly occurs in mitochondria where its membrane oxidative damage can cause mitochondrial dysfunction and TCA cycle-related enzyme dysfunction, leading to cuproptosis (152). First, Elesclomol-Cu^2+^ transports Cu^2+^ into and out of cells and copper ion channels solute carrier family 31 member 1 (SLC31A1) and ATP7B also regulate the accumulation of copper ions by mediating the entry and exocytosis of copper ions, respectively (Cai, et al., 2024). Intracellular Cu targets and binds to lipoylated mitochondrial enzymes in the TCA cycle such as dihydrolipoamide S-acetyltransferase (DLAT), inducing the aggregation of these proteins (18). The aggregation of these Cu-bound lipoylated mitochondrial proteins and the subsequent reduction of Fe–S (iron–sulfur) clusters lead to proteotoxic stress and ultimately cell death. Ferredoxin 1 (FDX1) and lipoic acid synthetase (LIAS) are the critical regulators of protein lipoylation, facilitating the aggregation of mitochondrial proteins and loss of Fe–S clusters (17, 18). Interestingly, genetic knockout of either FDX1 or LIAS leads to an accumulation of pyruvate and α-ketoglutarate, reducing protein lipoylation and inhibiting Cu-induced cell death (18), as shown in **Figure 3**. However, the mechanisms that underlie Cu-induced cell death are poorly understood. Therefore, further research is needed to determine the specific molecular details and potential therapeutic interventions for cuproptosis (32, 153).

### 5.1 Cuproptosis in diabetes and diabetic cardiovascular complications

Copper is an essential metal micronutrient, and abnormal copper metabolism is associated with multiple diseases, including Menkes disease, cardiovascular disease, Alzheimer’s disease, inflammation disorders, and cancers (32, 154–156). A high oral intake of Zn would affect copper absorption and can cause a decrease in copper absorption. Therefore, the Cu/Zn ratio is a key index to predict the risk of diabetes (157). Moreover, clinical studies have shown that plasma copper concentrations are positively associated with impaired glucose regulation in T1D and T2D, as well as all-cause mortality in patients with diabetes (158–160). Copper may induce diabetes via impaired glucose regulation and cuproptosis-induced oxidative stress and disrupted mitochondrial function.

Cuproptosis is caused by oxidative stress and impaired mitochondrial function, while diabetes alters the metabolism of cardiomyocytes, which also leads to oxidative stress that can result in increased copper levels in the cells (22). High levels of copper have been positively correlated with ROS generation (161). Copper promotes ROS generation, insulin resistance, and diabetes occurrence, leading to the development of atherosclerosis (162). On the one hand, accumulation of catalytically active Cu^2+^ in the cardiac extracellular myocardial induces copper toxicity, which is proposed as an important catalyst of cardiovascular damage in diabetes (163, 164). Copper toxicity leads to protein oxidation, GSH depletion, lipid damage, and redox imbalance (165, 166), which could cause impairment of heart function and exacerbate CVDs such as ischemic heart disease, arrhythmia, and heart hypertrophy. On the other hand, cardiomyocytes have the highest number of mitochondria. Therefore, it is reasonable to assume the potential induction of cuproptosis (108). This elevated copper accumulation can initiate a cascade of events that ultimately trigger cuproptosis (17) and its diabetic complications such as CVDs.

AGEs are formed by irreversible nonenzymatic reactions between reducing sugars, such as glucose, and amino groups in proteins, lipids, and nucleic acids during diabetes mellitus and could promote cardiomyocyte death through calcium overload, oxidative stress or excessive autophagy (167–169). Moreover, AGEs play an important role in the pathogenesis of diabetic complications, especially cardiomyopathy (170). AGEs promote the upregulation of high-affinity copper transporter-1 by upregulating the transcription factors activating transcription factor 3 (ATF3)/transcription factor PU.1 (SPI1)/SLC31A1, thus increasing intracellular copper accumulation and inducing cuproptosis in DCM (171). In addition, increased cuproptosis in the heart of streptozotocin-induced T1D and db/db T2D mice has been reported (171). Therefore, cuproptosis is closely related to the pathogenesis of DCM, which is expected to become another unique angle to explore the pathogenesis of diabetic cardiovascular complications, including DCM.

Besides, previous studies have suggested that changes in serum copper concentration and their effects may involve regulation at the genomic level (172, 173). A bioinformatics study recently showed that four key genes HSDL2, BCO2, CORIN, and SNORA80E are involved in the development of cardiomyocyte cuproptosis in patients with diabetes mellitus-associated HF through effects on the immune system (174). Previous studies also showed that hyperactivation of the PI3K/Akt/FoxO1/LOXL2 pathway promotes the development of myocardial fibrosis in the cardiomyocytes of patients with diabetes (175). This finding may enable us to quickly identify potentially relevant pathogenic factors. However, further cell or animal experiments are required to verify.

### 5.2 Cadmium and cuproptosis in diabetic cardiovascular complications

As cuproptosis is a novel kind of programmed cell death, recently reported by Tsvetkov et al. in March 2022 and its underlying mechanism and potential role of different environmental toxicants, including heavy metals such as inorganic arsenic, cadmium, and lead on cuproptosis are still not well known. Based on the results discussed in this review, heavy metals, especially cadmium, induce oxidative stress, pancreatic β-cell dysfunction, hyperglycemia, and disrupt insulin secretion, which induces and exacerbates T1D and T2D. Hyperglycemia induces oxidative stress, which causes mitochondrial dysfunction and AGE production (169). AGEs play an important role in the pathogenesis of diabetic complications, especially cardiomyopathy. Excessive AGEs in diabetes promote the upregulation of copper importer SLC31A1 through ATF3/SPI1, thereby increasing intracellular copper accumulation in cardiomyocytes and disturbing copper homeostasis. This encourages the decline of Fe–S cluster protein (FDX1, LIAS) and decreases lipoylation of DLAT-aggravated mitochondrial dysfunction in cardiomyocytes resulting in myocardial dysfunction (171). Moreover, excess copper-induced oxidative stress can lead to glutathione oxidation and decreased glutathione conjugation, ultimately resulting in cuproptosis and cardiotoxicity (176, 177) (**Figure 3**).

Although the direct effects of cadmium on hyperglycemia and the initiation of diabetes and the indirect effects of cadmium to exacerbate diabetic cardiovascular complications via ROS and AGE production have been demonstrated, further studies are undoubtedly needed to understand the interaction between cadmium and copper homeostasis in cardiac cuproptosis. Experimental evidence also has shown that inhibiting cuproptosis can attenuate mitochondrial dysfunction and cardiac damage in diabetic animal models. For instance, copper chelators like Trientine (triethylenetetramine, TETA) and Tetrathiomolybdate (TTM) can inhibit copper overload (178). However, further research is needed to fully understand the precise mechanisms linking cardiac cuproptosis in diabetic patients to explore potential therapeutic interventions targeting this pathway.

## 6 Conclusions and perspectives

Diabetes is characterized by high blood glucose levels that result from insulin deficiency, in the context of β- cell dysfunction, insulin resistance, or both (1). Oxidative stress and inflammation play a key role in the pathogenesis and progression of diabetes-induced CVD, where the enhanced expression of inflammatory proteins or cytokines such as oxidative stress-related proteins are shown to serve as biomarkers for the occurrence of cardiovascular complications (59). Oxidative stress and apoptosis by multifactorial mechanisms are considered as the most influential forms of cadmium toxicity in humans (94). The potential roles of cadmium in exacerbating cardiovascular complications in individuals with diabetes are described in the current review (**Figure 1**).

Cadmium exposure has been linked to cardiovascular complications in individuals with diabetes, potentially inducing ferroptosis and cuproptosis. The studies discussed above have provided preliminary, important evidence indicating the potential roles of ferroptosis and cuproptosis in cadmium-induced or exacerbated CVDs and diabetic cardiovascular complications. These processes involve disruption of trace element homeostasis, oxidative stress, GSH depletion, GPX4 reduction, and inflammation. Besides, cadmium induces common conditions such as iron overload in cardiomyocytes, mitochondrial dysfunction, oxidative stress, and lipid peroxidation, which lead to ferroptosis and cardiovascular complications in the individuals with diabetes (139). Furthermore, the present results are indicated that iron dysregulation by cadmium contributed to cadmium-induced ferroptosis, and induced or exacerbated cardiovascular complications in individuals with diabetes. Collectively, these results indicate that ferroptosis is involved in cadmium-induced cardiovascular complications in individuals with diabetes and is regulated by the Nrf2, HO-1, and GPX4 axis as well as promoting mitochondrial ROS generation (**Figure 2**).

Cuproptosis is the most recently described form of cell death triggered by copper accumulation and results in oxidative stress and mitochondrial dysfunction (22). This elevated copper accumulation can initiate a cascade of events that trigger cuproptosis (17) and its diabetic complications such as CVD. AGEs are formed by irreversible nonenzymatic reactions between reducing sugars, such as glucose, and amino groups in proteins, lipids, and nucleic acids during diabetes mellitus and could promote cardiomyocyte death through calcium overload, oxidative stress, or excessive autophagy (169). Cadmium-exacerbated hyperglycemia in diabetes patients can enhance AGEs, which excessive AGEs may promote the upregulation of copper importer SLC31A1 through ATF3/SPI1, thereby increasing intracellular copper accumulation in cardiomyocytes and induce myocardial cuproptosis (171). Overall, cuproptosis plays an essential role in cadmium-induced or exacerbated cardiovascular complications through copper importer SLC31A1 and mitochondrial accumulation of ROS in individuals with diabetes (**Figure 3**).

Little is known about the epigenetic effects of cadmium-exacerbates cardiovascular complications in the individuals with diabetes. Further research is needed to investigate the epigenetic effects of cadmium to exacerbate diabetic cardiovascular complications. Furthermore, the studies discussed above have provided preliminary, important evidence indicating the potential roles of ferroptosis and cuproptosis in cadmium-induced or exacerbated cardiovascular complications in individuals with diabetes. Nevertheless, the underlying mechanisms of ferroptosis and cuproptosis in cadmium-induced or exacerbated cardiovascular complications such as CVDs, cardiomyopathy, and coronary heart disease are still unclear, and further studies are needed to elucidate this issue. Activation of AMPK and Nrf2 plays an important role in preventing cardiac ferroptosis and preserving cardiac function in T2D mice (151). Even though Nrf2 activation can be a novel ideal therapeutic strategy for alleviating cardiotoxicity and cardiac ferroptosis caused due to cadmium exposure in diabetic patients, further study is needed to confirm this.

The direct effects of cadmium on hyperglycemia and the initiation of diabetes and the indirect effects of cadmium to exacerbate diabetic cardiovascular complications via ROS and AGE production have been demonstrated, further studies are undoubtedly needed to understand the interaction between cadmium and copper homeostasis in cardiac cuproptosis. Besides, experimental evidence has shown that inhibiting cuproptosis can attenuate mitochondrial dysfunction and cardiac damage in diabetic animal models. Copper chelators like TETA and TTM can inhibit copper overload (178). Further research is needed to fully understand the precise mechanisms linking cardiac cuproptosis in diabetic patients to explore potential therapeutic interventions targeting this pathway.
